# Book Review Strategies of Adjuvant Therapy

**DOI:** 10.1054/bjoc.2001.1781

**Published:** 2001-05

**Authors:** M Middleton

**Affiliations:** Christie Hospital


*British Journal of Cancer* (2001) **84**(9), 1286–1287
© 2001 Cancer Research Campaign
doi: 10.1054/ bjoc.2001.1781, available online at http://www.idealibrary.com on IDEAL®

### Book Reviews

### Strategies of Adjuvant Therapy

Edited by John M Kirkwood, Martin Dunitz Publishers, 2000, 240 pp, £65.00. 1 85317 3177

Biological therapies are an accepted part of anti-cancer treatment despite displaying only limited efficacy in early trials, which were largely in the setting of metastatic disease. The recent publication of studies describing the effect of adjuvant interferon treatment for melanoma highlights the potential of these agents at an earlier stage of the illness. In addition, our increasing understanding of the mechanisms of action of such agents permits the identification of new targets for treatment and better elucidation of the patient population likely to benefit from intervention.

*Strategies in Adjuvant Therapy* describes the potential for development of adjuvant biological therapies in a number of tumour types via a series of monographs. These are authoritative updates of ongoing research efforts in their particular fields but not all fulfil the brief described in the title and preface to the book. This is particularly true of the chapter on lymphoma: although giving an exhaustive account of novel therapeutics and their intracellular effects scant attention is paid to their use in the adjuvant setting, although trials in advanced disease are described. The discussion of renal cell cancer is similarly guilty of a concentration on therapy for advanced, metastatic disease with only a token attempt to relate this to adjuvant therapy. Furthermore, major ongoing studies are not covered.

The division of the book in site-specific chapters means that common themes in the development of adjuvant biological therapies are not emphasized. A summary of the issues concerning therapeutic target identification, patient selection/staging and treatment evaluation might have been a useful addition to the text. The chapters on melanoma stand out in that they explicitly address these problems in developing new treatments, but it is unfortunate that these strategic considerations are not given greater prominence. Molecular staging has its own chapter, which describes concisely the state of the art and problems associated with the accuracy of current techniques, but does not compare these with conventional staging protocols or with developing techniques such as sentinel node sampling.

Despite these minor reservations this remains a comprehensive account of an area in which radical changes can soon be expected. There are useful summaries of work in common tumours such as lung, breast, colorectal and prostate, as well as less prevalent cancers. For the general oncologist this provides an accessible summary of current trends in the development of adjuvant therapies and a useful update on biological therapy for other stages of disease. There is also sufficient detail for the site specialist and an extensive list of references to facilitate further reading.

*Mark Middleton*
*Christie Hospital*

doi: 10.1054/ bjoc.2001.1782, available online at http://www.idealibrary.com on

### Atlas of Breast Cancer, Second Edition

Edited by Daniel F. Hayes, Mosby, London, 2000

I did not have the good fortune of seeing the first edition of this Atlas, but the second is impressive. This is an Atlas which is clearly different from others in the field in being truly comprehensive. Apart from being an excellent practical guide to the management of breast cancer it has a strong underpinning of the biological and statistical foundations on which our current understanding of the disease is based. As stated in the preface by the editor, Dr Daniel Hayes, the idea of this Atlas germinated during discussions and teaching sessions at the breast unit of Dana-Farber Cancer Institute, and the reader will immediately recognize this connection from the choice of topics and the practical manner in which these are presented. The book comprises of 13 chapters and has approximately 100 pages with numerous beautifully coloured illustrations and tables which are easy to comprehend. In addition to Dr Hayes, there are 6 contributors but the book has been carefully edited with a clear flow of ideas.

The Atlas starts with an introductory chapter on natural history of breast cancer and is followed by one on risk factors, epidemiology, and development of breast cancer. The biological aspects of initiation, promotion and metastasis are beautifully depicted with illustrations. The diagram illustrating linkage analysis which led to the discovery of the familial breast cancer genes is particularly attractive and explicit. The general aspects of oncogenes and tumour suppressor genes are also presented in pictorial format. The third chapter is devoted to normal anatomy and development of the breast followed by one on breast cancer prevention. Here, the age-related risk profiles of different groups of women is shown in graphic format which the reader will find informative. The current status of breast cancer prevention has been rightfully presented with caution. The chapter on imaging has an American slant with screening mammography being recommended starting age 40.

The chapters on breast cancer surgery and processing of breast biopsies will be particularly useful for those training to become breast cancer surgeons. The techniques of breast conserving surgery and breast reconstruction are well illustrated. The methodologies for the detection of oestrogen receptor and c-erbB-2 oncogene over-expression/amplification are pictorially depicted for the benefit of those unfamiliar with these techniques. Benign disorders and pathology of breast cancer are explained with the use of beautiful and representative photomicrographs. The chapter on radiotherapy is excellent with clear diagrammatic representation of practical issues such as margin status after lumpectomy and factors that predict risk of local failure. The management of positive margins is very successfully presented in a picture format. In many places in the clinical chapters relevant figures from the

